# Mortality due to oral and oropharyngeal cancer in Uruguay from 1997 to 2014

**DOI:** 10.1590/1678-7757-2019-0166

**Published:** 2019-11-15

**Authors:** Maria Laura Cosetti-Olivera, Amanda Ramos da Cunha, Taiane Schaedler Prass, Marco Antonio Trevizani Martins, Fernando Neves Hugo, Manoela Domingues Martins

**Affiliations:** 1 Universidade Federal do Rio Grande do Sul, Programa de Pós-Graduação em Odontologia, Porto Alegre, Rio Grande do Sul, Brasil.; 2 Universidad de la República, Facultad de Odontologia, Departamento del Patologia y Estomatologia, Montevideo, Uruguay.; 3 Universidade Federal do Rio Grande do Sul, Departamento de Estatística, Porto Alegre, Rio Grande do Sul, Brasil.

**Keywords:** Mortality, Oral cancer, Oropharyngeal cancer, Tongue cancer

## Abstract

Oral and oropharyngeal cancer is considered a public health problem in several countries due to its high incidence and mortality rate.

**Objective:**

This study aimed to analyze oral and oropharyngeal cancer mortality in Uruguay from 1997 to 2014 by age, sex and country region.

**Methodology:**

A time series ecological study using secondary data was performed. Data on mortality due to oral and oropharyngeal cancers were obtained from the Vital Statistics Department of Uruguay's Ministry of Public Health.

**Results:**

The cumulative mortality rate due to oral and oropharyngeal cancer over the study period was of 19.26/100,000 persons in women and 83.61/100.000 in men, with a mean annual rate of 1.75/100,000 in women and 7.60/100,000 in men. Mortality rate from both sites during the study period was 4.34 times higher in men than in women. Malignant neoplasms of other parts of the tongue and base of tongue showed the highest mortality rate. The means of the annual coefficients of deaths were higher for the age groups between 50 and 69 years. Higher mortality rates of oral and oropharyngeal cancer were observed in Artigas (4.63) and Cerro Largo (3.75).

**Conclusions:**

Our study described a high mortality rate for oral and oropharyngeal cancer in Uruguay from 1997 to 2014. According to the country's health department, men, tongue cancer, and oral cavity had higher mortality rates, with some variation. Prevention strategies with control of risk factors and early diagnosis are necessary to improve survival in the Uruguayan population.

## Introduction

Head and neck squamous cell carcinoma (HNSCC) is in the group of malignant tumors originated in the oral cavity, oropharynx, hypopharynx, and larynx.^1^ According to the International Agency for Research on Cancer (IARC), HNSCC affected more than half a million people in 2012, and, of these, 300,373 cases were oral squamous cell carcinoma (OSCC).^2^ An estimate of 325,000 deaths have been annually attributed to HNSCC around the world.^3^

OSCC is considered a serious public health problem in several countries due to its high incidence and mortality rate.^1^^,^^4^ Several factors increase the OSCC development risk and seem to act by increasing the rates of cellular mutation. Exogenous factors related to lifestyle, especially tobacco and alcohol consumption, seem to be particularly important. HPV infection is usually associated with oropharyngeal cancer as well.^5^

Uruguay is a South American country, which is home to 3,286,314 million people and 43% of the population live in the capital, Montevideo.^6^ The National Cancer Program of Uruguay published their survey of cancer (2007 to 2011) and showed that oropharyngeal cancer is the seventh most common cancer in the country. It showed a higher incidence in men (n=1,079 cases) and a rate of 10.33 cases *per* 100,000 men and 2.76 cases *per* 100,000 women. In the capital, 468 cases occurred in men, equivalent to 11.37 *per* 100,000 men, and 201 cases in women, accounting for 2.99 cases *per* 100,000 women.^7^ A study performed by Oliveira, et al.^8^ (2015) analyzing the profile of the primary oral squamous cell carcinoma (OSCC) in Uruguay found that the male:female ratio was 3.8:1, the average age was 60.75 years (±11.26) for the population studied, and 42.5% of the cases occurred on the tongue. The study concluded that OCCS diagnosis in Uruguay is at late stages, with a poor prognosis and low survival rate.

In Uruguay between 2004-2008, the mortality rate for oral and pharynx cancer was 6.46/100,000 in men and 1.16/100,000 in women.^9^ Until now, no study has been completed in Uruguay analyzing both sites for a long period of time. Also, understanding the behavior of the disease over time is important to guide public health policies directed towards screening, to monitor the results of therapies used and establish epidemiological surveillance and control of oral and oropharyngeal cancer. Studies that specifically analyze these regions are non-existent in Uruguay. Thus, this study aimed to assess the rate of oral and oropharyngeal cancer mortality in Uruguay and its rates by age, sex, and regions of the country.

## Methodology

This is a descriptive ecological study using secondary data. Data on mortality due to oral and oropharyngeal cancer between 1997 and 2014 were obtained from the Vital Statistics Department of Uruguay's Ministry of Public Health. The year 2011 was not analyzed, since no data was available due to problems in the national register. The data about sociodemographic factors of the Uruguayan population were obtained from the National Statistics Institute of Uruguay, in 2011. This study was approved by the Research Ethics Committee of the School of Dentistry of the University of the Republic, Uruguay (358/16).

The data analyzed in this study included deaths caused by oral and oropharyngeal cancers. The anatomical sites of the neoplasms were determined by the International Classification of Diseases, according to the codes used in the 10^th^ revision (ICD-10) and have been in use since 1994. This study included Categories C00 to C10:

C00 – Malignant Neoplasm of Lip;
C01 – Malignant Neoplasm of Base of Tongue;
C02 – Malignant Neoplasm of Other and Unspecified Parts of Tongue;
C03 – Malignant Neoplasm of Gum;
C04 – Malignant Neoplasm of Floor of Mouth;
C05 – Malignant Neoplasm of Palate;
C06 – Malignant Neoplasm of Other and Unspecified Parts of Mouth;
C07 – Malignant Neoplasm of Parotid Gland;
C08 – Malignant Neoplasm of Other and Unspecified Major Salivary Glands;
C09 – Malignant Neoplasm of Tonsil and
C10 – Malignant Neoplasms of the Oropharynx

Uruguay has 19 regions or provinces: Artigas, Canelones, Cerro Largo, Colonia, Durazno, Flores, Florida, Lavalleja, Maldonado, Montevideo, Paysandu, Rio Negro, Rivera, Rocha, Salto, San Jose, Soriano, Tacuarembo and Treinta y Tres. This study analyzed mortality trends in each of them.

All mortality rates (number of deaths related to oral and oropharyngeal cancers *per* 100,000 inhabitants) analyzed and showed in this study were standardized by sex and age groups (15 to 19 years; 20 to 29 years; 30 to 39 years; 40 to 49 years; 50 to 59 years; 60 to 69 years; 70 to 79 years and 80 years or more), using the direct method, taking as reference the world population in 2011, adopting the “Dell STATISTICA” software package. Standardization was used with the objective of accounting for and removing the effects of factors related to the distribution of disease in the population that may interfere in the risk of mortality by oral and oropharyngeal cancer in the Uruguayan population (for example, sex distribution). Mean annual mortality rates represent the standardized arithmetic mean of annual mortalities rates from 1997 to 2014. All rates were multiplied by 100,000.

## Results

Between 1997 and 2014, 1,696 deaths from oral and oropharyngeal cancers were reported in Uruguay. The cumulative mortality rate due to oral and oropharyngeal cancer over the study period was of 19.26/100,000 persons in women and 83.61/100.000 men, with a mean annual rate of 1.75/100,000 in women and 7.60/100,000 in men.

The deaths were distributed into five categories, according to the anatomical site of the tumor: lip cancer (C00), tongue cancer (C01 and C02), oral cavity cancer (C03, C04, C05 and C06), salivary gland cancer (C07 and C08) and oropharyngeal cancer (C09 and C10). Table 1 shows the distribution of the deaths from 1997 to 2014, considering these five categories, and the ratio between the male and female crude mortality rates, for each category and total. The ratio between male and female rates was 3.23:1, the lowest ratio was for salivary gland cancer (1.83:1) and the highest ratio was for oropharyngeal cancer (6.40:1).

**Table 1:** Death distribution from oral and oropharyngeal cancer, according to the tumor anatomical site and sex of the subjects, Uruguay, 1997-2014

Anatomical site	Number of deaths (%)		F	M	Ratio
Lip cancer	32	(1.88)	7	25	3.57
Tongue cancer	664	(39.06)	157	507	3.23
Oral cavity cancer (excluding tongue and lip)	410	(24.12)	114	296	2.60
Salivary gland cancer	198	(11.65)	70	128	1.83
Oropharyngeal cancer	392	(23.06)	53	339	6.40
Total	1,696	(100)	401	1,295	3.23

The proportional distribution by anatomical site of the deaths from oral and oropharyngeal cancers showed that malignant neoplasms of other and unspecified parts of the tongue (C02) represented 25.88% of cases, followed by malignant neoplasms of the base of the tongue (C01), with 13.18%. Malignant neoplasm of the gum (C03) and malignant neoplasm of other and unspecified major salivary glands (C08) showed the lowest mortality rates.

For each anatomical site, the absolute and relative frequencies of deaths from 1997 to 2014 and the mean of the annual coefficients adjusted for sex and age (annual mortality rate *per* 100,000 inhabitants) are presented. The oral cavity has been considered a site that included other parts of the tongue, gums, floor of the mouth, palate, other parts of the mouth and salivary glands. In is this study, all oral cavity sub-sites represented 1,080 (63.70%) of deaths. Oropharyngeal cancer included the sub-sites, as described by WHO, such as the base of the tongue, tonsils and oropharynx and was responsible for 616 (36.30%) deaths (Table 2).

**Table 2:** Mortality by oral and oropharyngeal cancer, according to the anatomical site, Uruguay, 1997-2014. Absolute and relative frequencies of deaths and an average of the coefficients annually adjusted by sex and age (by 100,000 inhabitants)

Anatomical site	Number of deaths	%	Mean of the annual coefficients
Lip	32	1.88	0.05
Base of the tongue	224	13.18	0.43
Other parts of the tongue	440	25.88	0.78
Gums	9	0.53	0.02
Floor of the mouth	187	11.00	0.34
Palate	107	6.29	0.18
Other parts of the mouth	107	6.29	0.18
Parotid gland	186	10.94	0.30
Other major salivary glands	12	0.71	0.02
Tonsils	198	11.65	0.37
Oropharynx	194	11.41	0.36
Total	1,696	100.00	

Table 3 shows that the mean of the annual coefficients and the (cumulative) coefficients for the whole period, adjusted for sex and age group (*per* 100,000 inhabitants), for females and males and the proportion between these (M/F) coefficients for the period. The mortality rate from oral cavity and oropharyngeal cancer for the study period was 4.34 times higher in men than in women. Considering each of the anatomical sites, the mean of M/F ratios for the period was 4.70. The only site where the rate was higher for females was site C08 (malignant neoplasms of other and unspecified major salivary glands), in which it was 0.94. All sites of oropharyngeal cancer (base of the tongue, tonsils, and oropharynx) had M/F rate up to nine (mean of 9.16). Oral cavity sites exhibited a lower M/F rate with a mean of 3.02.

**Table 3:** Mortality by cancers of the oral cavity and oropharynx, according to anatomical site and sex (F = Female, M = Male), Uruguay, 1997-2014

Anatomical site	F - Mean	M - Mean	F – Cumulative	M -Cumulative	M/F
Lip	0.02	0.09	0.31	1.48	4.83
Base of the tongue	0.08	0.77	1.43	13.11	9.15
Other parts of the tongue	0.37	1.18	6.34	20.14	3.18
Gums	0.01	0.02	0.16	0.38	2.43
Floor of mouth	0.13	0.56	2.15	9.52	4.44
Palate	0.09	0.26	1.56	4.46	2.86
Others parts of the mouth	0.09	0.27	1.51	4.67	3.09
Parotid gland	0.18	0.42	3.00	7.21	2.40
Other major					
salivary glands	0.02	0.02	0.38	0.35	0.94
Tonsils	0.07	0.66	1.20	11.26	9.36
Oropharynx	0.07	0.65	1.23	11.03	8.97
Total	–	–	19.26	83.61	4.34
Mean	–	–	1.75	7.60	4.70*

NOTE:

* This value refers to the average of the ratios M/F and not to the proportion of the average values of the coefficients for females (1.75) and males (7.60)

For the 20-29 age group, it was observed that no cases were reported before 2004. Table 4 shows the absolute and relative frequencies of deaths from 1997 to 2014 (excluding 2011, since that data was not available due to a failure in the database) for each age group and the average of the annual coefficients adjusted by sex and age group (per 100,000 inhabitants). The means of the annual coefficients were higher for the age groups between 50 and 79 years. The mean of the annual coefficients and the (cumulative) coefficients for the whole period, adjusted for sex and age group (per 100,000 inhabitants) was 2.74 in women and 11.94 in men for each age group and the proportion between these (M/F) coefficients for the period was 4.84. (Table 4). The mortality rates by oral and oropharyngeal cancers during the study period was 4.34 times higher in men than in women and the age group between 40-49 years had the highest M/F coefficient (10.88), followed by the 50-59 group (8.50).

**Table 4:** Mortality by oral and oropharyngeal cancer, for each group age, Uruguay, 1997-2014

Age Group	Deaths (%)		F – Mean	M – Mean	F – Cumulative	M – Cumulative	M/F
15 |- 20	1	(0.06)	0.01	–	0.09	–	–
20 |- 30	8	(0.47)	0.02	0.03	0.27	0.48	1.76
30 |- 40	16	(0.94)	0.02	0.06	0.42	0.98	2.34
40 |- 50	99	(5.84)	0.04	0.47	0.73	7.94	10.88
50 |- 60	365	(21.52)	0.18	1.51	3.02	25.72	8.50
60 |- 70	490	(28.89)	0.27	1.53	4.52	25.97	5.75
70 |- 80	407	(24.00)	0.27	0.92	4.58	15.68	3.43
80 |-	310	(18.28)	0.33	0.40	5.63	6.84	1.22
**Mean**	**1.696**	**(100)**			**2.74**	**11.94**	**4.84**

Regarding the distribution for regions in the country, the time series of annual coefficients, adjusted by sex and age group (annual mortality rate *per* 100,000 inhabitants) from 1997 to 2014, showed a decrease in the rates in Artigas and Rivera in the total population and in men. In addition, in Flores, periods of high rates alternated with periods of zero rate. The absolute and relative frequencies of deaths in the study period, for each region and the average of the annual coefficients adjusted by sex and age group (*per* 100,000 inhabitants), showed that mean annual coefficients of deaths vary from 2.09, in Rocha, to 4.63 in Artigas. Montevideo represented 44.10% of the deaths in the period, but the average annual mortality rate for this region was 3.21 deaths *per* 100,000 inhabitants. Montevideo is where approximately half of the country's population lives. This means that for the whole (cumulative) period, adjusted by sex and age group (*per* 100,000 inhabitants) are shown in Table 5, which revealed a value of 16.72 in women and 79.82 in men, making the male/female proportion of 5.22. Artigas showed the highest M/F ratio (12.53) followed by Cerro Largo (10.01), the lowest ratio was in Florida (2.24), where the cumulative coefficient in women was 31.90 and in men was 71.35.

**Table 5:** Mortality by oral and oropharyngeal cancer, according to department and sex (F = Female, M = Male), Uruguay, 1997-2014

Department	F – Mean	M – Mean	F – Cumulative	M – Cumulative	M/F
Artigas	0.69	8.59	11.66	146.02	12.53
Canelones	1.22	4.96	20.70	84.24	4.07
Cerro Largo	0.68	6.82	11.58	115.95	10.01
Colonia	0.89	3.80	15.16	64.55	4.26
Durazno	0.96	3.53	16.30	59.98	3.68
Flores	1.03	4.75	17.54	80.78	4.61
Florida	1.88	4.20	31.90	71.35	2.24
Lavalleja	1.09	4.73	18.45	80.40	4.36
Maldonado	0.74	4.81	12.56	81.76	6.51
Montevideo	1.26	5.16	21.41	87.69	4.10
Paysandu	0.98	4.43	16.71	75.35	4.51
Rio Negro	1.05	5.06	17.84	86.07	4.82
Rivera	0.66	4.05	11.24	68.88	6.13
Rocha	0.96	3.22	16.28	54.68	3.36
Salto	1.12	4.02	18.99	68.41	3.60
San Jose	0.68	5.01	11.61	85.20	7.34
Soriano	0.73	3.47	12.45	58.92	4.73
Tacuarembo	1.02	5.07	17.34	86.24	4.97
Treinta y Tres	1.06	3.54	18.03	60.14	3.34
**Total**	**–**	**–**	**16.72**	**79.82**	**5.22**

## Discussion

To understand the mortality rate due to oral and oropharyngeal cancers for a population may influence new strategies to improve prevention and promote early diagnosis policies for these diseases. This study is the first one that assess, over a long period of time, the mortality rate by oral and oropharyngeal cancer in Uruguay according to sex, age, anatomic site, and geographical distribution. In general, our main findings reveal higher mortality rates in cases of oral cavity cancer compared to oropharyngeal cancer. Tongue cancer affecting men who live in the capital of the country showed the highest rates of mortality in the study.

Our results found that of 1.696 deaths registered in the study period, the oral cavity (63.70%) was more affected than the oropharynx (36.30%). This differs from the study published by Perea, et al.^10^ (2018), which revealed more deaths due to pharyngeal cancer than oral cavity cancer in Brazil, but when tonsils and oropharyngeal (except for the base of the tongue) cancers were considered, the average was similar in both regions. In Germany, Hertrampf, et al.^11^ (2015) reported that 54% of deaths were related to pharyngeal cancer, 40% to oral cavity cancer and 6% due to salivary gland cancer. Explaining the differences among our results and other studies is difficult, but one possible hypothesis is that Brazil and Germany have more effective strategies for oral cancer prevention or early diagnosis that impact the mortality rates for pharyngeal cancer.

We observed that tongue cancer was related to higher rates of mortality, since 440 cases of cancer were reported in the oral cavity and 224 cases in the oropharynx. These results agree with the data described by Oliveira, et al.^8^ (2015), which found that, in Uruguay, oral cancer is more prevalent on the tongue and its diagnosis tend to be at a later stage. Other studies also showed a higher mortality for tongue cancer, varying between 20% to 50% of cases.^12^^,^^13^ Several studies suggested that lack of oral examinations, late diagnosis and advanced stage of the diseases were the main causes of higher mortality rates associated with tongue tumors.^14^^–^^15^ Moreover, tongue tumors usually cause early metastases in the lymph nodes, promoting an increase in the mortality rate.^16^

The death rates between men and women have been studied extensively.^17^^–^^20^ Most countries estimate oral and oropharyngeal cancer mortality to be 3-4 *per* 100,000 men and 1.5-2 *per* 100,000 women at age-adjusted rates, and studies often indicate that women have a higher survival rate than men.^1^^,^^21^ Our results showed that deaths from oral and oropharyngeal cancers in Uruguay during 1997-2014 were 4.34 times greater in males than in females. These findings confirm previous studies worldwide.^1^^,^^17^^,^^19^^,^^22^ However, our male-female ratio was slightly higher than the ratio of other studies conducted in the region; Chile, for instance, varies between 2.8:1 and 2.3:1.^22^^,^^23^ In addition, correlating theses aspects with other risk factors such as tobacco and alcohol consumption in Uruguay is important. In 2014, the National Statistical Institute of Uruguay reported that 27.0% of men over 15 years old use tobacco compared to 17.9% of women.^24^ The May 2014 report from the National Cancer Registry indicated that the rates observed between 1990 and 2012 increased in both sexes, and this increase is considered significant in women.^7^ Perea, et al.^10^ (2018) suggested that another explanation for the differences between the sexes is that women tend to have regular dental appointments, which can improve their earlier diagnosis of oral cancer.

Analyzing the mortality related to an anatomical site found an increase up to a 9.0 in the M/F death ratio in sites like the base of the tongue, tonsils, and oropharynx. This modification can be associated to risk factors. Although our study did not assess risk factors associated to oropharyngeal cancer, it is important to discuss that besides tobacco and alcohol exposure, other aspects such as HPV infection and *mate* consumption have been reported. HPV infection has been associated to epidemiological and mortality rate modification in oropharyngeal cancer, especially in tonsils.^25^^–^^26^ An increase in incidence and an improved prognosis of HPV-positive tumors has been reported.^27^ In addition, *mate* consumption, which is a common habit in Uruguayan population, have been associated to an increased risk for oral cancer. It probably occurs due to the combination of the beverage temperature and its chemical composition.^28^ New studies involving the analysis of all risk factors should be performed to better analyze their connection with oropharyngeal cancer epidemiology and mortality.

This research identified differences among country regions. The higher mortality rates of oral and oropharyngeal cancer were observed in Artigas (4.63) and Cerro Largo (3.75). These results are in accordance with the study carried out by Barrios, et al. in Uruguay for the period of 2004-2008 that showed that Artigas, Cerro Largo y San Jose had mortality rates of up to 9/100,000 in men, while in women the highest rates were in Salto y Treinta y Tres.^9^ However, our results revealed that the mortality rates in Artigas and Rivera were higher, but they have been decreasing during the 17 years of our study evaluation time. Both regions are bordering the southern portion of Brazil and could be exposed to Brazilian policies on cancer prevention and early diagnosis strategies. Perea, et al.^10^ (2018) analyzed trends for oral and oropharyngeal cancer in all macro-regions of Brazil, finding an overall decrease in the South and Southern regions, possibly due to their anti-smoking policies. The development of preventive actions focusing on early detection and diagnosis in individuals at risk, particularly within the scope of primary health care, may be key in reducing mortality due to oral and oropharyngeal cancers.

This study is based on secondary information data registered with the National Statistics Institute of Uruguay. This type of study design has limitations because it is dependent on the accuracy and completeness of available information systems. Another limitation is related to the study design. Ecological studies are useful for raising hypotheses; however, they are not able to provide information about causality. According to IARC-WHO evaluation of registry data worldwide, the registry tracking the incidence of cancer in Uruguay is a high quality instrument, while mortality information is classified as medium quality registration data.

## Conclusions

Our results found that mortality due to oral cancer is higher than oropharyngeal cancer. In both sites, tongue cancer, men, and adults between 50 and 69 years old showed higher mortality rates. Improving the control of risk factors, mainly tobacco and alcohol, investigating HPV-status and implementing strategies for prevention and early diagnosis of these lesions is important.
